# Corticotropin-releasing factor and GABA in the ventral tegmental area modulate partner preference formation in male and female prairie voles (*Microtus ochrogaster*)

**DOI:** 10.3389/fnins.2024.1430447

**Published:** 2024-07-23

**Authors:** Kyle Richard Gossman, Camryn Serra Lowe, Adrianna Kirckof, Sydney Vanmeerhaeghe, Adam Steven Smith

**Affiliations:** ^1^Department of Pharmacology and Toxicology, University of Kansas, Lawrence, KS, United States; ^2^Program in Neuroscience, University of Kansas, Lawrence, KS, United States

**Keywords:** prairie voles, ventral tegmental area, CRF, GABA, pair bond, partner preference

## Abstract

**Introduction:**

The mesolimbic reward system is associated with the promotion and rewarding benefits of social relationships. In the socially monogamous prairie vole (*Microtus ochrogaster*), the establishment of a pair bond can be displayed by a robust preference for a breeding partner and aggressive rejection of unfamiliar conspecifics. Mesolimbic dopamine signaling influences bond-related behaviors within the vole through dopamine transmission and receptor activity in the nucleus accumbens. However, only one experiment has examined how the ventral tegmental area (VTA), a region that produces much of the fore- and mid-brain dopamine, regulates these social behaviors. Specifically, inhibition of either glutamate or GABA neurons in the VTA during a brief courtship promoted a partner preference formation in male prairie voles. The VTA is a heterogeneous structure that contains dopamine, GABA, and glutamate neurons as well as receives a variety of projections including corticotropin-releasing factor (CRF) suggested to modulate dopamine release.

**Methods:**

We used pharmacological manipulation to examine how GABA and CRF signaling in the VTA modulate partner preference formation in male and female prairie voles. Specifically, we used a 3 h partner preference test, a social choice test, to assess the formation of a partner preference following an infused bicuculline and CRF during a 1 h cohabitation and muscimol and CP154526, a CRFR1 antagonist, during a 24 h cohabitation with an opposite-sex conspecific.

**Results:**

Our study demonstrated that bicuculline, a GABA_*A*_ receptor antagonist, and CRF in the VTA promoted a partner preference, whereas low-dose muscimol, a GABA_*A*_ receptor agonist, and CP154526, a CRFR1 antagonist, inhibited a partner preference in both male and female prairie voles.

**Conclusion:**

This study demonstrated that GABA and CRF inputs into the VTA is necessary for the formation of a partner preference in male and female prairie voles.

## 1 Introduction

The functional significance of social attachment in humans, including pair bonding between partners and parental care toward offspring, has been documented cross-culturally (Jankowiak and Fischer, 1992; Young et al., 2011). Paired individuals, particularly those in stable marital relationships, have been shown to live longer compared to their unpaired counterparts, a finding noted across demographic groups (House et al., 1988; Lillard and Waite, 1995). High levels of intimacy between pairs have also been correlated inversely with negative psychological states, such as depressed moods, and positively with immune function and cardiovascular health (Waltz et al., 1988; Kiecolt-Glaser and Newton, 2001). Thus, intimate relationships represent significant aspects of our social world (Yamaguchi et al., 2015). It is critical that social partners display “commitment” through context-appropriate behaviors, such as selective affiliation to a current partner and rejection (or aggression in some species) of potential partners. Classically, the mesolimbic reward system has been associated with motivation and assigning salience to situational cues. In addition, this network has been shown to regulate the rewarding aspects of social attachment (Aragona and Wang, 2009). Recent functional connectivity research has also demonstrated that the regions of the mesolimbic reward system cluster together or show similar neural activation with one another when interacting with a partner, which is lost when interacting with a stranger conspecific (Ortiz et al., 2018; Gossman et al., 2021). However, research studying social attachment mainly focuses on the activity of the anterior cingulate cortex and nucleus accumbens, which use the neurotransmitter dopamine to control the formation of attachment, reward, and processing of emotions (Nestler and Carlezon, 2006; Baik, 2013; Hostetler et al., 2017; Yin et al., 2018; Horie et al., 2020). Surprisingly, few studies have examined the role of the ventral tegmental area (VTA), a dopamine-rich source hub for the mesolimbic reward system, in regulating social attachment. However, models that form these types of social attachments or pair bonds, as well as display distinct social behaviors associated with these bonds are limited in animal research.

The prairie vole (*Microtus ochrogaster*) is a socially monogamous rodent that forms long-term social attachments or pair bonds between breeding pairs, thus providing a unique model to characterize the role the VTA has in the regulation of pair bond formation. The vole model has been utilized for more than three decades to study the neurobiology of pair bonding, which has led to well-defined behavioral characteristics of pair bonding and much of the current knowledge of neuromechanisms of these behaviors (Smith et al., 2013; Tickerhoof and Smith, 2017). Specifically, dopamine signaling in the nucleus accumbens has been shown to regulate pair bond-associated behaviors, such that activation of dopamine receptor 2 regulates pair bond formation (i.e., partner preference) and dopamine receptor 1 regulates pair bond maintenance (i.e., aggression) (Gingrich et al., 2000; Aragona et al., 2006). Although dopamine has been well established in the regulation of pair bond formation, there is currently only one study that has focused on the VTA, a dopamine-rich nucleus of the mesolimbic system, during pair bond formation in prairie voles (Curtis and Wang, 2005). This study showed that inhibition of either glutamate or GABA neurons promotes a partner preference formation in male prairie voles after a 6 h cohabitation, a length of time insufficient for male voles to form a partner preference (Curtis and Wang, 2005). This study suggests a local circuit within the VTA—glutamate neurons project to and stimulate GABA neurons which project to and inhibit dopamine neurons. Thus, inhibition of glutamate or GABA could disinhibit dopamine neurons allowing for greater VTA dopamine release to downstream target sites. While the VTA is a heterogeneous structure that contains glutamate and GABA neurons that modulate dopamine neurons, the VTA also receives a variety of inputs such as corticotrophin-releasing factor (CRF) that are suggested to stimulate dopamine release (Morales and Margolis, 2017; Cai and Tong, 2022). Specifically, it has been estimated that there is CRHR1 on approximately 98% of dopamine-expressing neurons in the VTA (Refojo et al., 2011; Zalachoras et al., 2022). Thus far, one study demonstrated that the CRF into the nucleus accumbens accelerated a partner preference in male prairie voles (Lim et al., 2007); however, it has yet to be shown if CRF into the VTA has an influence on pair bond formation and maintenance in prairie voles. Thus, this study looks to further our understanding of how the local circuit and neurochemical inputs into the VTA influence partner preference formation in prairie voles. Here, we use pharmacological manipulation in the VTA to assess how GABA and CRF modulate partner preference in male and female prairie voles.

## 2 Materials and methods

### 2.1 Animals

Male and female subjects were captive-bred descendants from populations captured in southern Illinois. Voles were weaned on postnatal day 21 and housed with a non-sibling, same-sex conspecific in cages (29.2 L × 19.1 W × 12.7 H cm) which contained corn cob bedding and crinkle paper nesting material. Voles had access to food (LabDiet Rabbit Diet 5321) and water *ad libitum*. Colony rooms were kept on a 14L:10D photoperiod (Lights on at 0600 h) and at a temperature range of 21 ± 1°C. The subjects were sexually naïve, 60–90 days old at the beginning of the experiment. All procedures were conducted in accordance with the National Institutes of Health Guide and Use of Laboratory Animals and the Institutional Animal Care and Use Committee and the University of Kansas.

### 2.2 Stereotactic surgery

One week prior to pairing with an opposite-sex conspecific, subjects were anesthetized with an intraperitoneal injection of ketamine/dexmedetomidine cocktail (75/1 mg/kg) in sterile saline. Subjects were then head-fixed into a stereotaxic instrument (Stoelting) and a small incision was made to expose the skull. The skull was leveled using bregma and lambda as reference points. Once the head was aligned, holes were drilled, bilaterally, to target the VTA (ML: ± 0.79, DV: −4.91, AP: −3.20). Stereotaxic coordinates were validated with the use of the Allen Mouse Brain Atlas. Guide cannulae (26-gauge, Cat #: 62129, RWD Life Science) were fixed to the skull with a surgical screw, ethyl-2-cyanoacrylate-based adhesive, and dental cement. Once, the dental cement was set, a dummy cannula (RWD Life Scienc)e was placed in the guide cannula, and a cap was placed over the top of the dummy and guide cannulae (RWD Life Science).

### 2.3 Drug preparation

The drugs used in this experiment included bicuculline (GABA_*A*_ receptor antagonist, Cat #: ALX-550-515-M050, Lot #: 03072218, Enzo), muscimol (GABA_*A*_ receptor agonist, Cat #: 2763-96-4, Lot #: 3938537, EMD Millipore), CRF (Cat #: C3042, Lot #: 166977, Sigma), and CP154526 (CRFR1 antagonist, Cat #: 2779, Lot #: 3B1265193, Tocris). All drugs were aliquoted in either ddH_2_0 (bicuculline), artificial cerebrospinal fluid (aCSF; 150mM Na^+^, 3.0 mM K^+^, 1.4mM Ca^2+^, 0.8 mM Mg^2+^, 1.0mM P^3–^, 155mM Cl−) (CRF), or DMSO (0.025% or 0.0025% muscimol and 0.05% or 0.1% CP154526) at a 1 mg/ml concentration. On the day of testing, drug stocks were diluted to reach the appropriate working concentration in aCSF.

### 2.4 Site-specific drug administration

Thirty minutes prior to cohabitation, 200 nL aCSF containing no drug (aCSF), 5 ng bicuculline, 0.1 pg CRF, 5 ng (low dose) or 50 ng (high dose) muscimol, or 10 pg (low dose) or 100 pg (high dose) CP154526 was bilaterally injected into the VTA with a 1mm projection using a 33-gauge internal cannula (RWD Life Science) through the guide cannula. The rate of infusion was 200 nl/min, which was controlled with an infusion pump (KD Scientific). Following the infusion, the internal cannula was left for an additional minute before retracting it from the brain. After infusions, the subjects were returned to their home cage. The drug doses were selected based on previous literature using these drugs in prairie voles and other rodent models (Laviolette et al., 2004; Curtis and Wang, 2005; Lim et al., 2007; Iyilikci et al., 2016). Brains were harvested after behavioral testing for histological validation of cannula placement. Partial or complete histological misses were removed from the data analysis.

### 2.5 Partner preference test (PPT)

Thirty minutes after infusions, subjects were paired with a reproductively-sterilized, novel opposite-sex conspecific for either a 1 h or 24 h cohabitation (DeVries et al., 1995; Bales and Carter, 2003; Aragona and Wang, 2004). Female partners were estrogen primed with daily subcutaneous injection of estradiol benzoate at 1 μg/100 μL sesame oil for three consecutive days (Carter et al., 1988; Amadei et al., 2017; Gossman et al., 2021). Subjects that received either bicuculline or CRF were paired for 1 h, and subjects that received either muscimol or CP154526 were paired for 24 h. A partner preference test (PPT) was used, which is a well-documented behavioral assessment of pair bonding in prairie voles (Williams et al., 1992; Beery, 2021). In short, PPT utilizes a three-chambered arena (75 L x 20 W x 25 H cm) with a layer of corn cob bedding. The two stimulus animals, which include the partner of the subject and a novel opposite-sex conspecific, were tethered to the outer chambers. The female stimulus animals were estradiol benzoate primed as described above. The subject was restricted to the center chamber until the behavioral assessment began. Once the test began, the subject was allowed access to all chambers for a total of 3 h. Behavioral testing was completed between 1200 and 1600 h. Duration of stationary contact (i.e., side-by-side contact plus huddling) between the subject and either stimulus voles and the number of chamber crosses by the subject were recorded. All behavioral tests were manually scored by an experimenter blinded to experimental conditions using Solomon Coder (andraspeter.com).

### 2.6 Statistical analysis

Three-way mixed-model ANOVAs were used to assess the effects of drug condition, biological sex of subject, and social stimulus on stationary contact duration (side-by-side contact plus huddling) and chamber crosses during the PPT. Bonferroni post-hoc comparisons were used to determine significant differences between conditions when ANOVAs included significant main effects or interactions. Effects were considered significant at *p* < 0.05.

## 3 Results

### 3.1 Bicuculline or CRF intra-VTA injection before short-term cohabitation increased partner preference behavior

**FIGURE 1:**
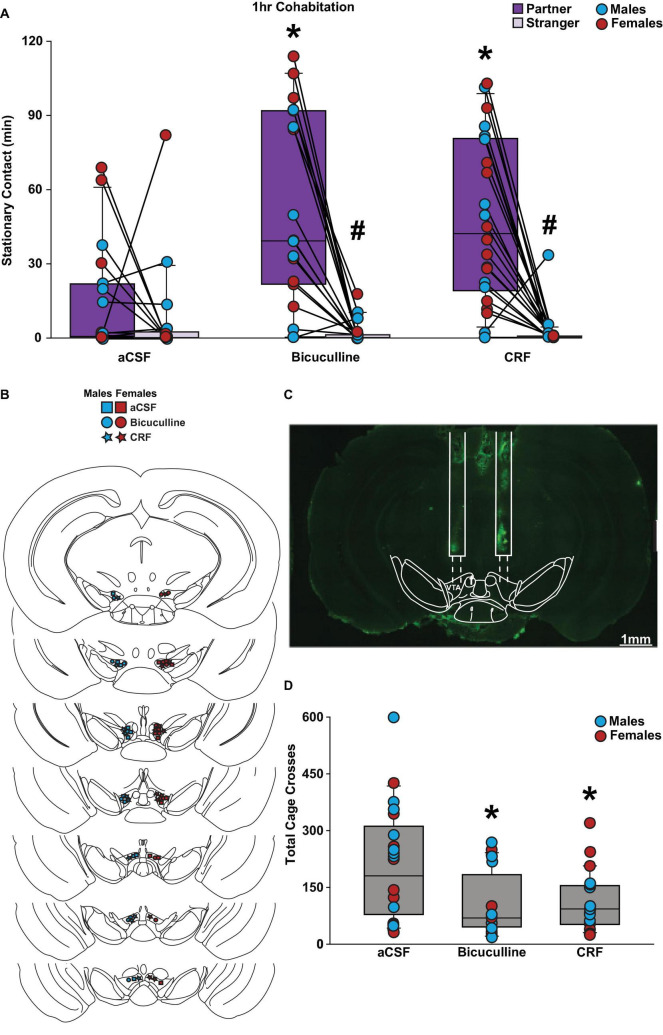
Bicuculline and CRF treatments promoted a partner preference in male and female prairie voles after a 1 h cohabitation. Stationary contact refers to the total of side-by-side and huddling. **(A)** Male and female voles infused with bicuculline (*n* = 9–10 per sex) and CRF (*n* = 10–12 per sex) demonstrated a significant preference toward their partner compared to a novel opposite-sex conspecific. Bicuculline and CRF groups exhibited significantly higher stationary contact time with their partner compared to the artificial cerebrospinal fluid (aCSF) (*n* = 9–11 per sex) group. **(B)** Placement of successful stereotactic hits that were included in the data analysis. **(C)** Representative image of cannula placement. **(D)** The aCSF group demonstrated an increase in total cage crosses comparted to both bicuculline and CRF groups. Three-way mixed-model ANOVA with Bonferroni post-hoc were used to analyze behavioral data. #*p* < 0.05 vs. social stimulus. *p < 0.05 vs. aCSF. No sex differences were observed.

There were main effects for social choice (partner vs stranger, *F*_(1,55)_ = 52.698, *p* < 0.001) and drug conditions (*F*_(2,55)_ = 5.900, *p* < 0.005). Moreover, there was a significant interaction between social choice and drug conditions (*F*_(2,55)_ = 8.651, p < 0.001). Specifically, subjects injected with bicuculline or CRF in the VTA before a 1 h cohabitation with a new partner spent an increased amount of time in stationary contact with their partner compared to a stranger vole (Figure 1A). However, control-treated voles did not differ in contact time with either stimulus vole. Also, overall stationary contact was higher in the bicuculline and CRF-treated voles compared to the voles in the control (aCSF) group (*p* < 0.001). Validation of guide cannula was confirmed for each subject in the pharmacological groups (Figures 1B, C). Lastly, the aCSF group had more total cage crosses compared to the drug-treated groups (*F*_(2,58)_ = 6.164, *p* = 0.004; Figure 1D). This is likely due to the decrease in overall stationary contact time in this group compared to the drug conditions. There were no main effects or interactions with biological sex of the subject observed for any of the statistical analyses. Altogether, these data suggest that inhibition of GABA_A_ receptors and activation of CRF receptors in the VTA was sufficient to promote a partner preference, as well as promote overall social interaction in both male and female prairie voles.

### 3.2 Mucismol or CP154526 intra-VTA injections before long-term cohabitation inhibited partner preference behaviors

**FIGURE 2:**
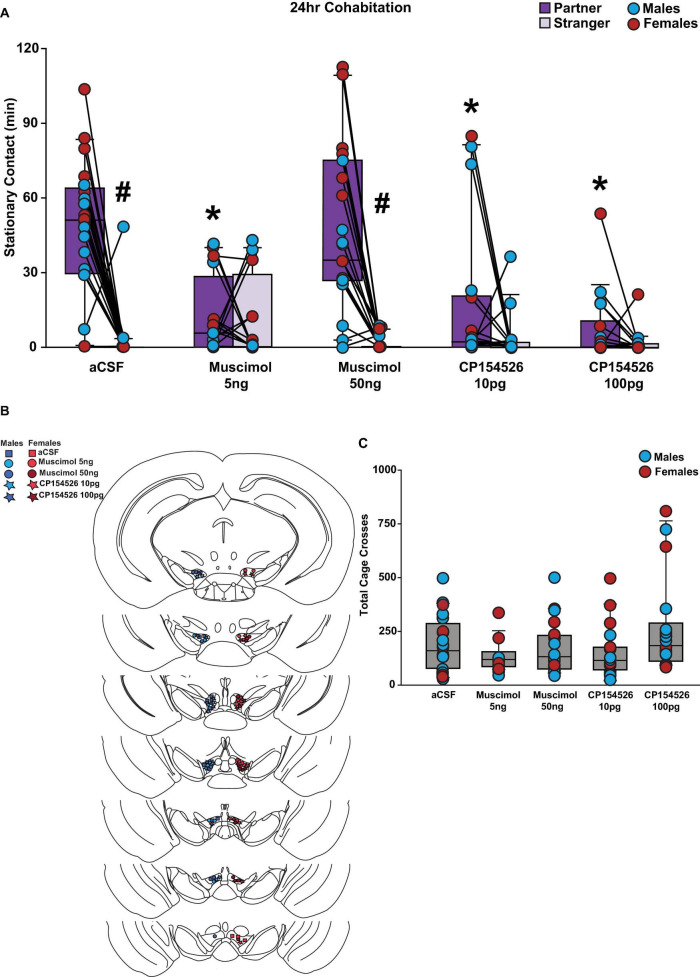
Low-dose muscimol and both low and high dose CP154526 inhibited a partner preference in male and female prairie voles after a 24 h cohabitation. Stationary contact refers to the total of side-by-side and huddling. **(A)** Male and female voles infused with low (5 ng) dose muscimol (*n* = 8 per sex) and low (10pg) or high (100pg) dose CP154526 (*n* = 8–10 per sex) did not exhibit a significant partner preference. However, subjects infused with high (50ng) dose muscimol (*n* = 9–10 per sex) or artificial cerebrospinal fluid (aCSF) (n = 9–11 per sex) demonstrated a significant preference toward their partner compared to a novel opposite-sex conspecific. Low dose muscimol and low or high dose CP154526 treated voles exhibited significantly lower stationary contact time with their partner compared to the aCSF and high dose muscimol groups. **(B)** Placement of successful stereotactic hits for the low or high dose muscimol, CP154526, and aCSF groups that were included in the data analysis. **(C)** Drug groups did not demonstrate a difference in total cage crosses when compared to the aCSF groups. Three-way mixed-model ANOVA with Bonferroni post-hoc were used to analyze behavioral data. #*p* < 0.05 vs. social stimulus. *p < 0.05 vs. aCSF. No sex differences were observed.

To demonstrate that GABA and CRF in the VTA are sufficient and necessary for partner preference formation in prairie voles, we infused muscimol, a GABA_A_ receptor agonist, or CP154526, a CRFR1 antagonist, into the VTA before a 24 h cohabitation with a new opposite-sex partner and a partner preference test was conducted after cohabitation. We observed main effects for social choice (partner vs. stranger, *F*_(1,80)_ = 62.893, *p* < 0.001) and drug conditions (*F*_(4,80)_ = 11.671, *p* < 0.001). There was a significant interaction between social choice and drug conditions (F_(4,80)_ = 12.160, *p* < 0.001). Specifically, control voles formed a preference for the new partner followed 24 h cohabitation; however, low dose muscimol inhibited a partner preference in both male and female prairie voles, but high dose muscimol did not. In addition, both low and high doses of CP154526 inhibited partner preference formation in male and female prairie voles (Figure 2A). Placement of the guide cannula was confirmed for each male and female subject (Figure 2B). There was no main effect for biological sex of the subject. There were interactions between sex and social choice (*F*_(1,80)_ = 7.091, *p* < 0.01) as well as sex and drug condition (*F*_(4,80)_ = 4.041, *p* = 0.005). However, post-hoc analyses revealed no group differences for either interaction. Therefore, no sex differences were observed. There were no differences between total cage crosses (*F*_(4,85)_ = 2.112, *p* = 0.086, Figure 2C).

## 4 Discussion

Social attachments, particularly those in long-term marriages and other romantic partnerships, present well-documented positive health benefits (House et al., 1988; Kiecolt-Glaser and Newton, 2001). One particular neural network thought to regulate various aspects of social attachment is the mesolimbic reward system (Aragona and Wang, 2009). However, there has been limited research to assess the role of the VTA, a dopamine-rich hub of the mesolimbic reward system, regulation of social attachment and commitment behaviors. Here we report that CRF promotes and GABA inhibits pair bond formation in both male and female prairie voles. Specifically, CRF and bicuculline administration in the VTA at the start of a short-term (1 h) cohabitation with a new partner facilitated the formation of a partner preference in males and females. Conversely, muscimol and CP154526 injections in the VTA at the start of a long-term (24 h) cohabitation with a new partner blocked the formation of a partner preference. These pharmacological effects were observed in both male and female prairie voles suggesting that, in a stress-naïve state, CRF signaling into the VTA may be appetitive and facilitates a pair bond while GABA signaling is aversive and hinders such social attachment.

Consistent with research from Curtis and Wang (2005), our study demonstrated that VTA GABA inhibited partner preference in male prairie voles. We also included females in our study and documented for the first time that VTA GABA inhibits partner preference formation in female prairie voles. Surprisingly, bicuculline was able to promote a partner preference in male and female prairie voles after a 1 h cohabitation compared to a 6 h cohabitation used in the previous study, as it has been well-documented that female prairie voles require 6–12 h of cohabitation and male prairie voles need a 24 h cohabitation to form this social preference (Aragona and Wang, 2004; Beery, 2021). There are limited studies that examine how VTA GABA neurons regulate the rewarding aspects and motivation for social attachment behaviors. One study proposed a circuit and demonstrated an increase in the activity of the GABAergic pathway from the medial preoptic area to the VTA during maternal behaviors including hovering, crouching, and body licking (Caba et al., 2019). Another study demonstrated that activation of GABA, by the use of muscimol, in the VTA decreased pup retrieval, increased the latency to retrieve all the pups, and decreased both nursing duration and crouch duration (Numan et al., 2009). Similar to our study, these studies suggest that the activation of VTA GABA, during a short-time period (i.e., 15 to 60 mins), in social attachments such as pair bonding and mother-infant bonds, regulate the reward aspects and motivation for social interaction and the display of attachment behaviors. Apart from social attachments, VTA GABA neurons have been shown to regulate a variety of behaviors in response to reward and stress (Bouarab et al., 2019). Stimulation of VTA GABA has been shown to regulate reward consumption and increase aversive behaviors in mice (van Zessen et al., 2012). Also, VTA GABA interneurons have been suggested to regulate and encode the expectation of reward and reward outcomes (Pan et al., 2013; Eshel et al., 2015). Beyond reward, there is an increase in the activity of VTA GABA neurons during stressful experiences, including foot shock or a looming stimulus (Tan et al., 2012; Zhou et al., 2019). VTA GABA neurons also project throughout the mesolimbic reward system, particularly the nucleus accumbens (Bouarab et al., 2019). GABA projections from the VTA to the nucleus accumbens regulates associative learning by acting on cholinergic interneurons and inhibition of these neurons rescues reward-seeking and reward reinforcement (Creed et al., 2014; Al-Hasani et al., 2021; Lowes et al., 2021). Together, this work identifies a role for VTA GABA in reward, stress, and motivation, however our study is one of the first to expands the role of VTA GABA function in regulating social attachment. Further studies should be conducted to examine how VTA GABA regulates the coding of a new partner as a rewarding and motivating stimulus during social attachment formation and maintenance behaviors.

We also demonstrated that CRF administration in the VTA facilitated partner preference formation in male and female prairie voles. Thus far, CRF system is well-known and is particularly studied in stress-related behaviors (Hostetler and Ryabinin, 2013). In the VTA, CRF is suggested to regulate behaviors associated with stress and addiction. CRF in the VTA specifically regulates social stress, mediates cocaine seeking, and modulates food reward in rats as well as controls aversive effects and nicotine intake in mice (Blacktop et al., 2011; Wanat et al., 2013; Grieder et al., 2014; Holly et al., 2016). CRF inputs into the VTA also act on VTA GABA neurons. Specifically, two studies have shown CRF modulates GABA neurons to mediate stress-induced ethanol and cocaine seeking (Blacktop et al., 2016; Harlan et al., 2018). Recently, however, studies have shown that CRF can promote appetitive motivation and positive valence in a stress naïve state. Specifically in the limbic system, CRF microinjections into the nucleus accumbens can promote partner preference formation, cue-triggered motivation for sucrose, conditioned place preference, and DA release in stress naïve males (Pecina et al., 2006; Lim et al., 2007; Chen et al., 2012; Lemos et al., 2012). A few studies in the prairie vole have shown similar effects of CRF through other routes of administration, including intracerebroventricular and site-specific nucleus accumbens injections (DeVries et al., 2002; Lim et al., 2007; Bosch et al., 2009). In addition, CRF receptor antagonist into the nucleus accumbens has been shown to decrease overall sociability (DeVries et al., 2002). Beyond pair bonding, CRF in the extended amygdala and hypothalamus modulates maternal behaviors including maternal neglect and stress in rats (Klampfl et al., 2014, 2016, 2018). However, our study is one of the first to document that CRF signaling in the VTA promotes social attachments, specifically pair bonding, in prairie voles in a stress-naïve state. Given that VTA CRF signaling can produce appetitive or aversive effects, more research will be required to determine the mechanism of these effects, which will likely be stress-dependent.

Social interactions and attachments have many rewarding aspects that are suggested to be processed through the mesolimbic reward system, and in particular, regulated by the neurotransmitter dopamine. As the VTA is a dopamine-rich region, this would suggest that our pharmacological manipulation of GABA and CRF have either local projections to or directly modulate dopamine neurons that would project downstream to other regions of the mesolimbic reward system. VTA GABA projections to dopamine neurons have been a well-established local circuit in certain motivational and learned behaviors (Creed et al., 2014; Yang et al., 2018; Ohta et al., 2022; Song et al., 2024). However, it also has been shown that different GABA and dopamine populations in the VTA regulate specific memory activities, as well as VTA dopamine projects to other regions such as the basolateral amygdala during positive experiences, suggesting that different circuits can be activated during varying behaviors and these circuits should be examined during social attachment behaviors (Sun et al., 2021; Glykos and Fujisawa, 2023). Like GABA, CRF can directly modulate VTA dopamine release but also has indirect effects on dopamine through glutamate and GABA (Wanat et al., 2008; Wise and Morales, 2010; Refojo et al., 2011; Boyson et al., 2014; Zalachoras et al., 2022). Although dopamine has been shown to regulate motivation and rewarding behaviors and prairie vole pair bond formation specifically (Aragona et al., 2003, 2006; Liu and Wang, 2003; Lee and Beery, 2021), it remains uncertain how and if GABA and CRF directly modulate VTA dopamine neurons to promote a partner preference. Further investigation should be completed to investigate this potential circuit.

In conclusion, GABA and CRF in the VTA exhibit a novel and critical role in the formation of a partner preference in male and female prairie voles. This elucidates a particular local circuit in the VTA that may increase the positive valence coding of a new social partner, promoting a choice toward a partner over another novel social stimulus after a short cohabitation. With the rewarding benefits of social attachments, this would suggest that GABA and CRF act on VTA dopamine neurons to promote dopamine downstream; however, these circuits need further attention. Taken together, these findings further demonstrate how a region within the mesolimbic reward system modulates social attachment behaviors as well as provide future directions to examine how neurochemical systems interact with one another to modulate such behaviors.

## Data availability statement

The original contributions presented in the study are included in the article/supplementary material, further inquiries can be directed to the corresponding author.

## Ethics statement

The animal study was approved by the National Institutes of Health Guide and Use of Laboratory Animals and the Institutional Animal Care and Use Committee and the University of Kansas. The study was conducted in accordance with the local legislation and institutional requirements.

## Author contributions

KG: Conceptualization, Data curation, Formal analysis, Project administration, Validation, Writing−original draft, Writing−review and editing. CL: Data curation, Writing−review and editing. AK: Data curation, Writing−review and editing. SV: Data curation, Validation, Writing−review and editing. AS: Supervision, Conceptualization, Formal analysis, Funding acquisition, Resources, Writing−original draft, Writing−review and editing.
